# The Genome Russia project: closing the largest remaining omission on the world Genome map

**DOI:** 10.1186/s13742-015-0095-0

**Published:** 2015-11-13

**Authors:** Taras K. Oleksyk, Vladimir Brukhin, Stephen J. O’Brien

**Affiliations:** 1 Theodosius Dobzhansky Center for Genome Bioinformatics, St. Petersburg State University, St. Petersburg, Russia; 2 University of Puerto Rico at Mayaguez, Mayaguez, Puerto Rico

## Abstract

We are witnessing the great era of genome exploration of the world, as genetic variation in people is being detailed across multiple varied world populations in an effort unprecedented since the first human genome sequence appeared in 2001. However, these efforts have yet to produce a comprehensive mapping of humankind, because important regions of modern human civilization remain unexplored. The Genome Russia Project promises to fill one of the largest gaps, the expansive regions across the Russian Federation, informing not just medical genomics of the territories, but also the migration settlements  of historic and pre-historic Eurasian peoples.

## Background

Mapping the unabridged pattern of human genetic variation across the world represents one of the greatest exploration projects since the genomics era began in 2001 with a published draft of the human genome. Driven by the availability of samples and by technological advancements in next generation sequencing in the last decade, whole-genome sequencing has scaled up sequencing personal individual genomes of some audacious scientists (Drs. Venter and Watson) to carrying out entire global surveys of individual genomes, best represented by the 1,000 Genome project [1, 2].

In the three years since the first 1,000 Genomes consortium paper on human diversity was published, attention has shifted to national population genome projects. These include, for example, the 100,000 UK Genome Project, the Asian Genome Project, the Chinese Million Genomes endeavor, the African Genome Sequence Variation project, as well as whole-genome sequence population studies in the Netherlands, Qatar, Turkey, and Japan [3]. All of these projects serve as a major global reference resource for human genetic variation and provide a new roadmap and power for disease variant discoveries. However, all of these projects still make for an incomplete genome map of humankind.

**Fig. 1:**
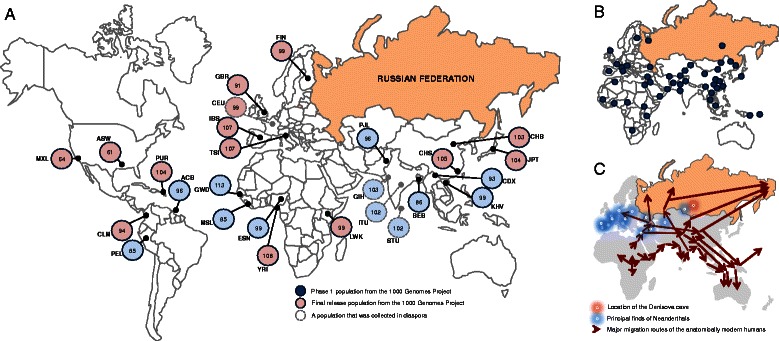
Distribution of publicly available genome sequences. **a** Worldwide locations of population samples with the whole genome data from the 1000 Genome Project [1]. Each circle represents the number of genome sequences publicly available at www.1000genomes.org. ASIA: BEB Bengali in Bangladesh; CDX Chinese Dai in Xishuangbanna, China; CHB Han Chinese in Bejing, China; CHS Southern Han Chinese, China; GIH Gujarati Indian in Houston,TX; ITU Indian Telugu in the UK; JPT Japanese in Tokyo, Japan; KHV Kinh in Ho Chi Minh City, Vietnam; PJL Punjabi in Lahore, Pakistan; STU Sri Lankan Tamil in the UK. AFRICA: ACB African Caribbean in Barbados; ASW African Ancestry in Southwest USA; ESN Esan in Nigeria; GWD Western Division, The Gambia; LWK Luhya in Webuye, Kenya; MSL Mende in Sierra Leone; YRI Yoruba in Ibadan, Nigeria; EUROPE: CEU Utah residents with Northern and Western European ancestry, USA; FIN Finnish in Finland; GBR British in England and Scotland; IBS Iberian in Spain; TSI Toscani in Italiy; THE AMERICAS: CLM Colombian in Medelin, Colombia; MXL Mexican Ancestry in Los Angeles, USA; PEL Peruvian in Lima, Peru; PUR Puerto Rican in Puerto Rico. Each circle represents the number of sequences in the final release. The dotted circles indicate populations that were collected in diaspora. **b** Eastern Hemisphere locations of population samples in surveys of worldwide genetic variation (HapMap, 1000 Genomes Project, Phase 1, and HGDP) [1, 4]. **c** Major human migration routes (adapted from [10]) and locations of other hominid remains out of Africa. The approximate locations of major Neanderthal and Denisovan finds are indicated by glowing circles

Looking at a world map showing these dynamic developments in genome sequencing, one cannot help but notice a great “wide gap” in the center (Fig. 1a): from the Baltic Sea to the Beringia Straits, Russia remains the largest vast swath of land, and people, for which the human genome landscape remains relatively unexplored. Note that even the larger population SNP array genotyping projects such as HGDP (~52 populations sampled worldwide) and the HapMap have little representation of ethnic groups in Russia (Fig. 1b, [1, 4]). Also, the European and East Asian population groups in the 1,000 Genome Project do not capture the rich background of genomic diversity in this part of the world — partly because of a difference in ancestry; partly because of its history of admixture (Fig. 1a). Recent population genetic studies of Russian indigenous populations have primarily employed mtDNA, STR, Y-chromosome haplogroups and genome SNP variants in certain regional ethnic populations, with little done on more comprehensive whole genome sequencing of Russian people (for citations see: http://genomerussia.bio.spbu.ru/?lang=en).

This is problematic given that the historic migratory milestones that founded modern Russian populations include the northward and westward expansion of the Indo-Europeans and the Uralic people, the westward expansion of the Turkic people, and centuries of admixture between them (Fig.1c). Further, the routes for peopling Northern and Central Europe inevitably led through this territory, then waves of great human migrations of recorded history pushed this way for centuries, followed by a known exchange of knowledge, and technology, and, likely, genes, along the Silk Road (Fig. 1c). These myriad migrations have created a complex patchwork of human diversity that is today’s Russia and somewhere hidden in Siberia reside the ancestors for modern Native Americans.

In the more distant past, gene exchange likely occurred between *Homo sapiens* and Neanderthal and Denisovan populations they encountered. The genetic contribution of the Neanderthal has not been well studied beyond Western Europe; nor has that of the Denisovan for South East Asia, despite their physical remains being unearthed in Siberia [5, 6]. Russian populations very likely contain ancestral components that aren’t easily found in the populations represented in the 1,000 Genomes or even in the comprehensive HGDP database. Hence, Russia needs a national genome project on its own.

With the current moderate cost of genome projects, a Russian centric project will likely be much less expensive than its predecessors, and it would bring numerous benefits to our understanding of population origins and disease on local, global and evolutionary scales, as detailed in Table 1.

**Table 1:** Six real benefits from genome Russia project to Russia, to science, and to the world genomics community

1. Low frequency and local variants that are discovered in population genome projects can be used to screen individuals with genetic disorders in genome wide association studies (GWAS), in clinical trials, and in genome assessment of proliferating cancer cells [1, 2]. Thus, Russian biomedical researchers will receive the benefit of an information resource that will build the baseline for future studies, including advances in precision/personalized medicine.
2. Russia has a history of population admixture, with the modern Russian population comprised of genetic contributions from three main ancestral ethnicities: European (Slavic, Baltic and Germanic), Uralic (Finno-Hungarian), and Altaian (Turkic), with the possible addition of traces from peoples that occupied the Eurasian Arctic and Siberia in the past (Fig. 1c). As yet, this genome admixture has not been well documented, and presents a new and unique opportunity to study population history in the wake of the great human migrations, the Black Death, the Great Silk Road diaspora, or recent demographic perturbations of the twentieth century.
3. An admixture history combined with the diverse environments faced by the local populations in Russia create a unique opportunity for disease gene discoveries through the use of mapping of admixture disequilibrium or admixture mapping [7]. This approach is known to be more powerful than a GWAS in homogeneous panmictic populations, and has been used to discover a number of health-related mutations in other populations (as per, for example, [8]). Given the difference in historic selection pressures, genome admixtures specific to Russia will contribute a wealth of new information bringing forth different risk and/or protective alleles that do not exist nor associate with disease, elsewhere in the world.
4. Studies of population ancestry and admixture in Russia would not be limited to modern humans. Recent reports have uncovered the exact details about when Neanderthals and modern humans interbred and have even suggested important disease-fighting genes derivative of those pre-historic encounters [6]. Much of the Neanderthal heritage may still be unaccounted for, as recent reports keep discovering new genes originating from this ancient admixture, and the spread of the Neanderthal is now documented as far as the Altai Mountains in Siberia [6]. The geographic source of Denisovan DNA is also Russian in origin, while its contribution is mainly found in Melanesia [5]. Given that most of the genetic landscape of Russia is little explored, we cannot state with any certainty that another great discovery is not hidden behind that great “wide gap” on the global genetic diversity map (Fig. 1a, b).
5. Thorough understanding of human migration and evolution requires a Russian genome project, given that the peopling of the Arctic and the American continents, came from ancestral populations in Russia, specifically those in Siberia. An analysis of the variety of populations in Russia should therefore provide key information about this stage of human migration.
6. Engaging Russia scientists and communities in an international project like this would help integrate its scientists into the world genomics community. The scientific output and training in Russia has diminished since the fall of the USSR in 1991 but the sustaining enormous intellectual potential has since become one of the world’s best secrets. Genome Russia will formally join the International 1,000 Genome project, with their thoroughly vetted and widely agreed ethical guidelines (www.1000genomes.org). Further, Genome Russia will be built upon the open release/access philosophy, a trend that is gaining momentum, but suspicion remains, as trust between Russia and Western governments has become challenged by the recent political exchanges [9].

The justifications for collecting, sequencing and analyzing populations from Russia in the immediate —rather than some distant— future, all impart the enormous significance that these populations have in the history of humankind and their value as a reservoir of knowledge about our health. Without filling the great “wide gap” on the genetic map of the world, we will remain handicapped in achieving our major goals for use of genomic information. The beginnings of such a Genome Russia Project are in fact being met with growing enthusiasm, as seen by its endorsement by the Russian Academy of Sciences and the Russian Ministry of Education and Science in a concerted effort to make it happen (http://genomerussia.bio.spbu.ru/?lang=en). While political diplomacies continue(9), the Genome Russia Project can and should become an example of international collaboration on the common ground and with the common goal of improving human health and betterment.

## Abbreviations

GWAS: Genome Wide Association Study
HGDP: Human Genome Diversity Project
mtDNA: mitochondrial deoxyribonucleic acid
SNP: Single Nucleotide Polymorphism
STR: Short Tandem Repeat
